# CBCT-to-CT Translation Using Registration-Based Generative Adversarial Networks in Patients with Head and Neck Cancer

**DOI:** 10.3390/cancers15072017

**Published:** 2023-03-28

**Authors:** Chitchaya Suwanraksa, Jidapa Bridhikitti, Thiansin Liamsuwan, Sitthichok Chaichulee

**Affiliations:** 1 Department of Radiology, Faculty of Medicine, Prince of Songkla University, Songkla 90110, Thailand; 2 Department of Biomedical Sciences and Biomedical Engineering, Faculty of Medicine, Prince of Songkla University, Songkla 90110, Thailand; 3 Princess Srisavangavadhana College of Medicine, Chulabhorn Royal Academy, Bangkok 10210, Thailand; 4 Research Center for Medical Data Analytics, Faculty of Medicine, Prince of Songkla University, Songkla 90110, Thailand

**Keywords:** radiotherapy, cone beam computed tomography, deep learning, image reconstruction

## Abstract

**Simple Summary:**

Cone-beam computed tomography (CBCT) not only plays an important role in image-guided radiation therapy (IGRT) but also has the potential for dose calculation. Because CBCT suffers from poor image quality and uncertainties in the Hounsfield unit (HU) values, the accuracy of dose calculation with CBCT is insufficient for clinical use. This study investigated deep learning approaches that utilize a generative adversarial network (GAN) with an additional registration network (RegNet) to generate synthetic CT (sCT) from CBCT. Our study addressed the limitation of having paired CT and CBCT with their anatomy perfectly aligned for supervised training. RegNet can dynamically estimate the correct labels, enabling supervised learning with noisy labels, whereas GAN learns the bidirectional mapping from CBCT to CT. The HU values for sCT were sufficiently accurate for dose calculation, while preserving the anatomy of CBCT with clear structural boundaries.

**Abstract:**

Recently, deep learning with generative adversarial networks (GANs) has been applied in multi-domain image-to-image translation. This study aims to improve the image quality of cone-beam computed tomography (CBCT) by generating synthetic CT (sCT) that maintains the patient’s anatomy as in CBCT, while having the image quality of CT. As CBCT and CT are acquired at different time points, it is challenging to obtain paired images with aligned anatomy for supervised training. To address this limitation, the study incorporated a registration network (RegNet) into GAN during training. RegNet can dynamically estimate the correct labels, allowing supervised learning with noisy labels. The study developed and evaluated the approach using imaging data from 146 patients with head and neck cancer. The results showed that GAN trained with RegNet performed better than those trained without RegNet. Specifically, in the UNIT model trained with RegNet, the mean absolute error (MAE) was reduced from 40.46 to 37.21, the root mean-square error (RMSE) was reduced from 119.45 to 108.86, the peak signal-to-noise ratio (PSNR) was increased from 28.67 to 29.55, and the structural similarity index (SSIM) was increased from 0.8630 to 0.8791. The sCT generated from the model had fewer artifacts and retained the anatomical information as in CBCT.

## 1. Introduction

Head and neck cancer represents one of the most common groups of malignant tumors worldwide, with approximately 830,000 new patients diagnosed annually and a mortality rate of 430,000 per year [1]. The major subsites of head and neck cancer include the oral cavity, pharynx, larynx, salivary glands, paranasal sinuses, and nasal cavity. Furthermore, more than 90% of these tumors are squamous cell carcinomas that respond well to radiation therapy.

### 1.1. Radiotherapy

The goal of radiation therapy is to maximize the dose to the tumor while minimizing it to surrounding organs at risk [2]. Curative radiotherapy for head and neck cancer requires the administration of high doses of radiation to a small area located very close to critical structures, such as the spinal cord, brainstem, brain optic pathways, brachial plexus, and salivary glands.

Thus, advanced radiotherapy treatments, such as intensity-modulated radiation therapy (IMRT), volumetric modulated arc therapy (VMAT), image-guided radiation therapy (IGRT), adaptive radiotherapy, and proton therapy, have been proposed to improve oncological outcomes, minimize radiation-induced toxicity, and broaden the scope of radiotherapy indications [3,4]. However, during the course of treatment, the patient’s anatomy may change from the day, so the computed tomography (CT) simulation was conducted due to the tumor’s response to radiation and residual motion. Radiotherapy treatment plans are generally unable to consider anatomical changes. Furthermore, if the treatment continues with the original treatment plan despite anatomical changes, the delivered dose will not be the same as the original planned dose. This may result in underdosing or overdosing of the target and organs at risk and may, in turn, affect the clinical outcome [5,6,7], particularly in proton therapy because it is more sensitive to variations in the patient anatomy than photon therapy. If the deviation between the planned dose and delivered dose is unlikely to be clinically significant, the treatment may continue with the original treatment plan. However, if the dose deviation is clinically significant, the physician may make the decision to continue or discontinue the treatment and establish a new plan [8,9].

One of the biggest current challenges is the development of an accurate method for assessing whether the dose deviation exceeds the tolerance level during the course of treatment. A CT simulator is the gold standard modality for treatment planning and dose calculation in radiotherapy. It produces a high-resolution three-dimensional image volume, referred to as planning CT (pCT), which demonstrates good geometrical accuracy and accurate Hounsfield unit (HU) values. The pCT has the CT number of each tissue correspond to the photon or proton attenuation inside the patient, which accounts for tissue heterogeneities. Thus, the pCT is primarily used to develop a treatment plan that is tailored to each patient [10].

In image-guided radiotherapy (IGRT), cone-beam computed tomography (CBCT) is most commonly used to verify the patient position before and after radiation delivery. It is not only useful for verifying the patient’s position but also has the potential to be used for dose calculation [11].

For each treatment session, the difference between the planned dose and the actual delivered dose can be observed via the dose calculation based on CBCT compared with that based on pCT. However, the HU values of CBCT may vary owing to beam hardening and scattered photons, which in turn may lead to incorrect dose calculation. Although hardware-based pre-processing methods, including a bowtie filter and anti-scatter grid, can prevent some of the scattered photons from reaching the detector, the image quality of CBCT remains inferior to that of pCT [12].

### 1.2. CBCT Image Quality Improvement

Several studies have investigated methods to increase the accuracy of CBCT-based dose calculation by changing HU values, such as HU overriding and CBCT calibration (with population-based CBCT, patient-specific CBCT, or phantom CBCT) [13,14,15]. However, these methods do not greatly improve image quality. Other studies have proposed methods for improving both image quality and dose calculation accuracy based on CBCT image post-processing, such as histogram matching [16] and image registration [17,18]. Poludniowski et al. [13] investigated a method for correcting CBCT numbers by removing scatter in the detector and using a patient-specific or phantom-based look-up table (LUT) to convert CBCT numbers to pCT numbers. They suggested that compared to pCT numbers, CBCT numbers with a MAE of less than 50 HU gave acceptable discrepancies in doses of less than 3%. Thing et al. [15] reported that dose calculation based on CBCT overestimated the re-planning CT dose, whereas dose calculation based on artifact-corrected CBCT improved the agreement between the CBCT dose and the re-planning CT dose. The main goal is to perform dose calculation using updated anatomical structures of the patient with sufficiently accurate HU values. Deformable image registration (DIR) is a method that is widely used in various clinical applications for radiotherapy, especially for dose calculation. It has been used to generate pseudo-CT images by distributing the HU from pCT to CBCT, thereby preserving the anatomical information acquired by CBCT. Giacometti et al. [19] evaluated the accuracy and efficiency of dose calculation in cancer patients based on CBCT using four methods: (i) standard CT calibration curve, (ii) CBCT site-specific calibration curve, (iii) HU override, and (iv) deformable registration. They found that deformable registration is the most accurate method for dose calculation based on CBCT images, although it still suffers from poor anatomical accuracy. DIR requires both CBCT and pCT with high image quality to achieve the best result. Kurz et al. [20] compared two methods, DIR and an intensity correction approach (population-based look-up table) for adaptive intensity-modulated photon (IMRT) and proton radiotherapy (IMRP) in head and neck cancer. They suggested that both methods are suitable for dose calculation in IMRT, but for IMRP, the DIR method is preferable. In addition to these two methods, scatter correction has also been proposed in various studies [21,22,23]. Kurz et al. [22] compared DIR and scatter correction in adaptive IMPT for prostate and head and neck cancer. They found that for patients with head and neck cancer, no considerable differences between the two methods were observed. However, for prostate cases, the DIR method resulted in incorrect contours, likely due to the more pronounced anatomical changes in the abdomen and the reduced soft-tissue contrast in the CBCT. Lalonde et al. [23] proposed scatter correction of CBCT images using deep convolutional neural networks for adaptive proton therapy of the head and neck. The results indicated that this approach achieved a level of accuracy comparable to Monte Carlo simulations while being significantly faster.

### 1.3. CT Synthesis Using Deep Learning

There has been a growing interest in using deep learning to generate sCT from CBCT. This is partly due to the increasing availability of high-performance computational resources over the last decade which has enabled the training of a large number of images, allowing fast image translation within a few milliseconds. Compared with traditional approaches, the use of deep learning to convert CBCT to sCT has resulted in significant improvements in image quality and speed. Ideally, sCT generated from CBCT should have CT numbers as accurate as pCT while maintaining anatomical accuracy similar to CBCT.

In general, medical image synthesis can be described as an image-to-image translation problem in which a model maps the source image (A) to the target image (B) [24]. Many models have been proposed for image-to-image translation, with the most popular being U-Net [25] and generative adversarial networks (GANs) [26]. U-Net is a U-shaped symmetric encoder-decoder architecture designed primarily for semantic segmentation. The encoder acts as a feature extractor and learns an abstract representation of the source image, whereas the decoder translates the feature representation into the target image. A GAN is a framework consisting of a deep generative model and a discriminative model through an adversarial process. The generator converts the source image into the target image, whereas the discriminator determines whether the target image is real or fake. Several network architectures are based on the GAN framework.

GANs are the most popular frameworks for CBCT-to-CT conversion. Certain studies have compared U-Net to GAN and reported that GANs show statistically better results [27,28], whereas others found slightly worse results [29]. Liang et al. [27] evaluated the image quality of sCT images generated from CBCT using deep learning for head-and-neck cancer patients. The authors evaluated U-Net and three GAN variants, including CycleGAN, DCGAN and PGGAN. U-Net performed significantly worse than any GAN variant. CycleGAN performed better than DCGAN and PGGAN in terms of image similarity and dose agreement. CycleGAN removed most of the scatter artifacts in CBCT images and the HU values of the resulting images were corrected, leading to an agreement of dose distributions with deformed pCT. The results suggested that CycleGAN can be effectively used to improve image quality before dose calculation in adaptive radiotherapy. Similar results were observed for CycleGAN by Kida et al. [30] in prostate cancer patients.

Several studies [28,29,31] investigated CBCT-to-CT translation using CycleGAN for multiple anatomical sites. Eck et al. [31] compared the image quality of sCT in the head and neck, thorax, and pelvis regions and reported the best results for the pelvic region. Maspero et al. [29] reported similar results for image similarity when they compared a single CycleGAN trained with all anatomical sites (head and neck, breast and lungs) and for individual CycleGANs trained independently for each anatomical site. Zhang et al. [28] trained GAN and tested it on CBCT acquired with a different CT scanner and a different anatomical site to demonstrate its ability to transfer pre-trained models to different anatomical regions and CT scanners.

Although certain discrepancies were still present in sCT, the deep learning approach resulted in a statistically significant improvement over CBCT. GAN variants have been well studied in both paired and unpaired training [27]; however, it remains unclear which variant is the best scheme for sCT generation. Several factors play important roles here, including data preparation, training scheme, network architectures, and loss functions. As for the data, the number of patients used to train the networks varies widely, ranging from 7 [32] to 205 [31]. A few studies have currently addressed the effects of increasing the training size. Increased image similarity with stable accuracy and stable losses was reported when up to 50 patients were trained [31,32,33]. However, the anatomical location and its variability between and within fractions may also influence the optimal number of patients to be included in a study.

Paired and unpaired training are two training schemes widely used in image translation. U-Net is trained with supervised paired training, whereas CycleGAN and UNIT have the option of using both unpaired and paired data for training. The condition for supervised paired training, that is, matching CBCT with pCT of the same geometry and slice, is clinically difficult. The first fraction of CBCT is geometrically close to pCT [31]; therefore, rigid or deformable registration can be used to determine the ground truth for paired training. However, the alignment may not be perfect because of respiratory motion or anatomical changes between acquisitions, especially in cases where the time between pCT and the first CBCT acquisition is more than a week. If the source and target images are misaligned, the errors will propagate through the model during training, which may cause unreasonable shifts in the translated images. In unpaired training, it is possible to train a model with CBCT (of any fraction) and pCT because the training data requirements are less demanding. This scheme works well with unpaired or misaligned images; however, its performance may not be ideal. The training process of unpaired data may be relatively unstable and may produce multiple solutions.

Recent advances in GANs for image translation have involved adopting adaptive registration into the model in order to account for imperfectly paired images (source and target images that may not be properly aligned). Arar et al. [34] were the first to demonstrate performance improvements in GANs for cross-modality image translation by adding a RegNet to either the input or output of the GAN, allowing the network to learn both translation and registration simultaneously. Kong et al. [35] introduced a RegNet to GANs to enable them to learn the misaligned noise distribution. This allowed the network to dynamically correct the noisy labels during training with the goal of finding a joint optimal solution for both image-to-image translation and image registration. The authors noted that incorporating a RegNet makes GANs insensitive to noise, which makes them a better choice for a wide range of scenarios, particularly for medical image-to-image translation tasks, where well-pixel-aligned data are not available. They demonstrated the effectiveness of this technique in MRI T1-T2 image translation. Yang et al. [36] investigated MRI cross-modality image translation, similar to Kong et al. [35], by incorporating a deformable registration method into GANs. Chen et al. [37] exploited a registration network to generate a deformation field that helps in improving GAN performance for multi-modal CR-MRI image translation. Although each study examined the incorporation of a registration method into GANs, they did so in slightly different ways. In this study, we adopted a registration-based image translation approach for radiotherapy, where we performed CBCT-to-CT image translation.

### 1.4. Study Objectives

This study investigated methods to improve the image quality of CBCT by generating sCT with the image quality of CT while preserving the patient’s anatomy as in CBCT. Because it is difficult to obtain CBCT with accurate anatomical alignment as in pCT, we applied RegNet to GANs so that it learns the distribution of misaligned noise to dynamically estimate the correct label during training. This makes supervised learning less restrictive and can help overcome the limitation that CBCT and pCT are not exactly paired. The purpose of this study was to investigate whether incorporating RegNet into GANs can lead to improved performance over conventional GANs.

Our study makes several contributions. First, we used RegNet with GANs in a supervised learning fashion to account for misaligned noise and generate sCT from CBCT, while most studies used unsupervised learning with unpaired datasets due to imperfect alignment of imaging data. Second, we demonstrated the effectiveness of the approach on a diverse dataset of over 146 patients with head and neck cancer and provided comprehensive results. Our findings suggest that RegNet enhances CBCT image quality compared to models without RegNet. Lastly, we showed the effectiveness of a hybrid model of GANs and variational autoencoders (VAEs) over the commonly used CycleGAN. The Hounsfield unit (HU) values for sCT were sufficiently accurate, while preserving the anatomy of CBCT with clear structural boundaries, indicating potential for dose calculation.

## 2. Materials and Methods

### 2.1. Clinical Dataset

This retrospective study was conducted on 146 patients with head and neck cancer using three variants of GANs: Pix2Pix, CycleGAN, and UNIT. The patients underwent radiotherapy using the Varian TrueBeam STx LINAC platform (Varian Medical Systems, Inc., Palo Alto, CA, USA) between January 2018 and December 2021 at Songklanagarind Hospital, Thailand. We included patients who underwent pCT with at least five CBCT fractions with a prescribed dose of >66 Gy. The exclusion criteria included the following: those who had palliative intent; those whose imaging data had the planning target volume (PTV), clinical target volume (CTV), brain stem and spinal cord outside the field of view (FOV) of CBCT, and those whose imaging data showed motion artifacts. This study was approved by the Office of Human Ethics Committee, Faculty of Medicine, Prince of Songkla University under approval number REC.64-004-7-2.

pCT images were acquired on a 16-row multidetector helical CT scanner with a tube voltage of 120 kV and a tube current of 350 mA. Its dimensions were 512×512 pixels with a voxel resolution of 1.14×1.14×3.00 mm^3^ (a slice thickness of 2–3 mm). CBCT was acquired on an on-board kV imager in the 1st fraction and was collected with a tube voltage of 110 kV, and a tube current of 40 mA. Its dimension were 512×512×93 pixels with a voxel spacing of 0.51×0.51×1.99 mm^3^.

Our dataset was divided into three sets: a training set of 95 patients, a validation set of 21 patients, and a test set of 30 patients. We trained an algorithm on the training set while tuning the model’s hyperparameters on the validation set, and evaluated it on the hold-out test set. Table 1 shows the patient characteristics for all sets, including sex, age, diagnosis site, pathology results, disease stage, and treatment. The disease stage was defined according to the 7th and 8th editions of the American Joint Committee’s TNM classification system as assessed by the treating physician.

During the acquisition of pCT and CBCT, the patients were placed in the supine position with the arms resting at the sides, the shoulders pulled down, and the neck in a neutral or extended posture on a special pillow. A thermoplastic mask was used to fix the position. In some cases, a bolus was placed on the mask to increase the dose to the skin for photon beams, and the patient was made to bite a polyvinylchloride (PVC) tube to separate the oral cavity. All patients were treated with a 6 MV photon beam at a dose rate of 600 monitor units per minute (MU/min) using the VMAT technique (2–4 arcs). The prescribed dose ranged from 66 to 70 Gy in 33 to 35 fractions. CBCT images were acquired on a Varian TrueBeam STx LINAC (Varian Medical System, Palo Alto, CA, USA) during the first three days, followed by weekly CBCT image acquisition for each patient.

### 2.2. Data Preparation

**Figure 1:**
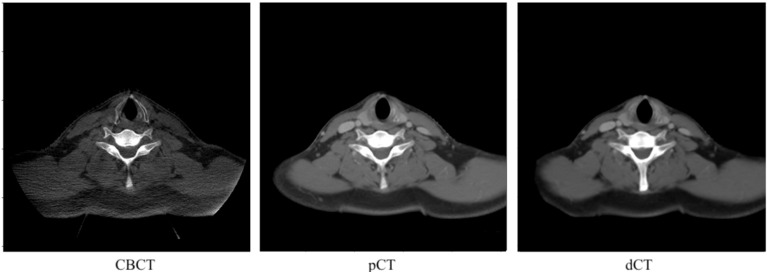
Examples of CBCT, pCT, and dCT images of the same slice.

**Figure 2:**
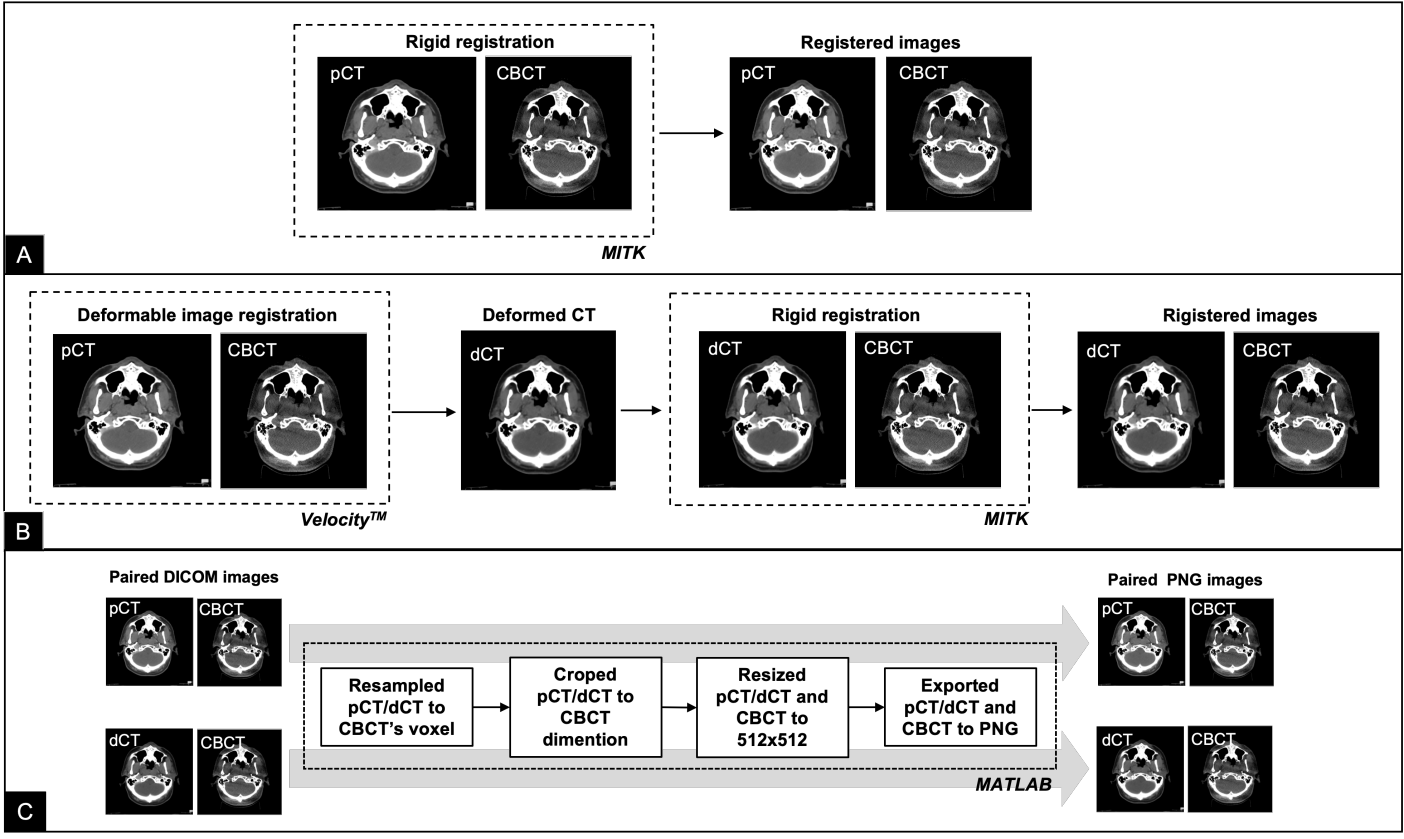
Flowchart of the data preparation process. (**A**) pCT was registered to CBCT using the multi-modal rigid registration algorithm with manual adjustment in MITK workbranch. (**B**) pCT was transformed to align with CBCT using the DIR algorithm in Velocity^™^. The HU values of pCT were then deformed to the CBCT anatomy to create dCT. dCT was then was registered to CBCT in MITK workbranch. (**C**) Both the pCT and dCT volumes were resampled to match the CBCT’s voxel spacing and cropped to match its dimensions using MATLAB.

The pCT and CBCT images were acquired at different time points and with different FOVs; therefore, neither image was aligned geometrically. pCT was registered to the first CBCT fraction using the multi-modal rigid registration algorithm with regular-step gradient descent for registration optimization and Mattes mutual information for similarity assessment using MITK Workbench v2018.04.2 (Deutsches Krebsforschungszentrum, Heidelberg, Germany), followed by a manual fine adjustment by experts. The pCT was also transformed to align with the first CBCT using the DIR algorithm in Velocity^™^ 4.1.4 software (Varian Medical System, Inc., Palo Alto, CA, USA). Image registration began with rigid registration using coordination from the online matching in the treatment room and was followed by deformable registration using the Elastic B-Spline model. In the overlap region, the HU from the pCT was deformed to the CBCT anatomy and used to create a new volume, referred to as the deformed CT image (dCT). Subsequently, both the pCT and dCT volumes were resampled to match the CBCT’s voxel spacing and then cropped to match the CBCT dimension using MATLAB R2020a (MathWorks, Portola Valley, CA, USA). All images were resized to 512×512 pixels and exported as 16-bit PNG images to preserve the original HU values. We discarded the first two slices and the last two slices of CBCT, pCT, and dCT as they were outside the CBCT’s FOV, resulting in 89 slices for each volume. Finally, the training set had 8455 slices (95 cases); the validation set had 1869 slices (21 cases); and the test set had 2670 slices (30 cases). Both the pCT and dCT were used as reference to train each deep learning model. Figure 1 shows examples of CBCT, pCT, and dCT images for the same slice. Figure 2 illustrates flow chart of the data preparation process.

As mentioned previously, the registration network (RegNet) is based on the work of Kong et al. [35]. The network is trained to acquire prior knowledge regarding the distribution of misaligned noise such that it can dynamically predict the deformation vector field (DVF), which in turn can be used to estimate the correct label, which is unknown in the real world. Our problem can be considered supervised learning with noisy labels. DVF describes the displacement of each pixel; thus, we can apply DVF to sCT to obtain the correct label.

**Figure 3:**
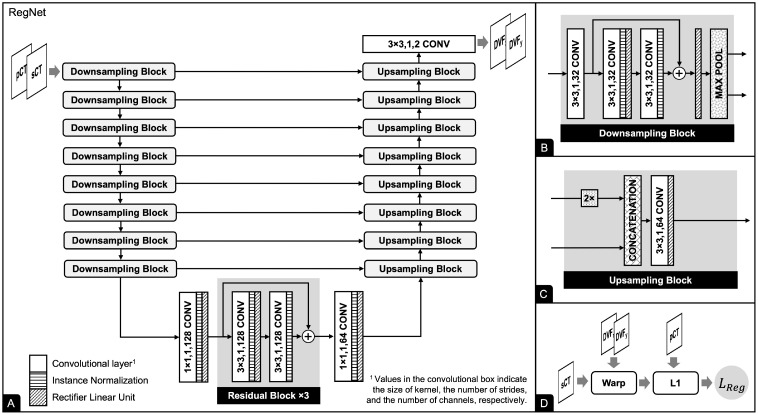
RegNet takes pCT and sCT and estimates deformation vector field (DVF). RegNet acts as a model for misaligned noise to correct the result, enabling supervised learning with noisy labels. (**A**) RegNet uses the U-Net framework consisting of eight downsampling blocks, three residual blocks, and eight upsampling blocks. (**B**) The downsampling block consists of a series of convolutions, residual connections, nonlinear activations, and max-pooling operations. (**C**) The upsampling block comprises a sequence of upsampling, concatenation, convolution, and nonlinear activation. (**D**) The schematic diagram of registration loss.

RegNet is implemented using U-Net, which uses both pCT acquired from CT simulation and sCT produced by the trained generator to predict DVF (see Figure 3). Our RegNet consists of eight downsampling blocks, three residual blocks, and eight upsampling blocks. The downsampling blocks extract features at different levels through a sequence of convolutions, residual connections, nonlinear activations, and max-pooling operations, whereas the upsampling blocks decode feature maps into DVF in horizontal and vertical dimensions through a sequence of upsampling, convolutions, and nonlinear activations. The DVF describes the displacement of each pixel, so we can apply DVF to sCT to obtain the correct result.

### 2.3. Registration Network

Given a training dataset ${\left\{\left(x_{n},{\tilde{y}}_{n}\right)\right\}}_{n=1}^{N}$ with *N* samples, in which $x_{n}$, ${\tilde{y}}_{n}$ are images from the source and target domains, respectively, *G* is a generator model, *R* is a RegNet model, and *T* is a spatial transformation function, the registration loss is calculated using the $L_{1}$ loss function:

(1) $$L_{Reg}=\frac{1}{N}\sum_{n=1}^{N}\left|{\tilde{y}}_{n}-T\left(G\left(x_{n}\right),R\left(G\left(x_{n}\right),{\tilde{y}}_{n}\right)\right)\right|$$

where $R(G\left(x_{n}\right),{\tilde{y}}_{n})$ results in DVF. Although minimizing $L_{Reg}$ leads to a better approximation of the DVF, it may result in a non-smooth DVF that may not be physically realistic [38]. A smoothness loss is applied to the DVF during training as a diffusion regularizer to encourage a smooth DVF:

(2) $$L_{Smooth}=\frac{1}{N}\sum_{n=1}^{N}|\delta R\left(G\left(x_{n}\right),{\tilde{y}}_{n}\right){)|}^{2}$$

### 2.4. Pix2Pix

**Figure 4:**
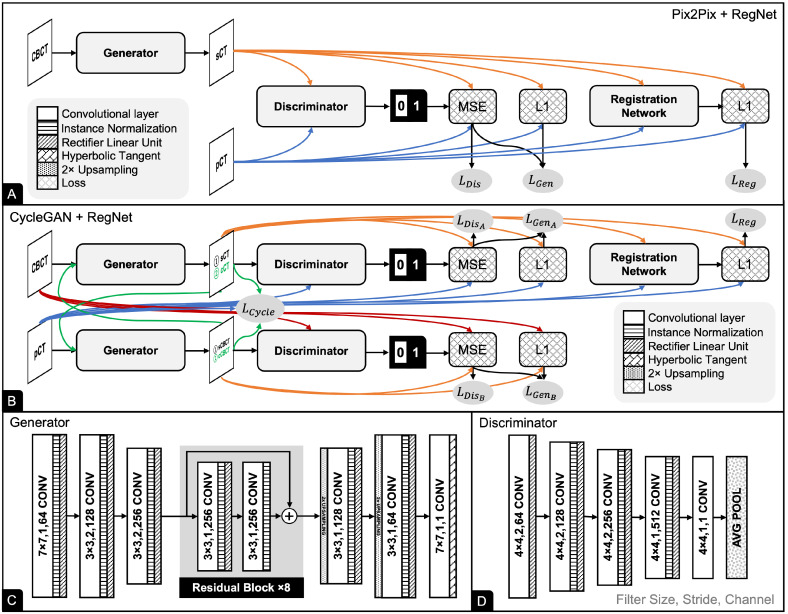
Pix2Pix and CycleGAN share common generator and discriminator architectures. RegNet was coupled with Pix2Pix and CycleGAN during training. (**A**) Pix2Pix has one discriminator and one generator. (**B**) CycleGAN has two discriminators and two generators. (**C**) The generator consists of three convolution blocks, eight residual blocks, and three convolution blocks with upsampling. (**D**) The discriminator comprises a series of convolutions, nonlinear activations, and average-pooling operations. Values in the convolutional layer box indicate the size of the kernel, the number of strides, and the number of channels, respectively.

Pix2Pix [39] is a GAN-based model that consist of a generator to generate sCT from CBCT and a discriminator to distinguish between sCT and pCT (see Figure 4A). The generator was implemented with a stack of eight residual blocks arranged in the encoder-decoder scheme (see Figure 4C). The discriminator was implemented using a series of convolutions, residual connections, nonlinear activations, and average-pooling operations, resulting in an output of 0 or 1 (see Figure 4D).

The output of the discriminator passes through a loss function that assigns low energy to the real images and higher energy to the synthetic images. Given a discriminator model *D*, the discriminator loss is defined as the mean square error (MSE) loss:

(3) $$L_{Dis}=\frac{1}{N}\sum_{n=1}^{N}{\left(1-D\left({\tilde{y}}_{n}\right)\right)}^{2}+\frac{1}{N}\sum_{n=1}^{N}{\left(0-D\left(G\left(x_{n}\right)\right)\right)}^{2}.$$

To preserve the HU values between the source and target images, a loss function for the generator is implemented using $L_{1}$ loss and MSE loss:

(4) $$L_{Gen}=\lambda (\frac{1}{N}\sum_{n=1}^{N}|{\tilde{y}}_{n}-G\left(x_{n}\right)\left|\right)+\frac{1}{N}\sum_{n=1}^{N}{\left(1-D\left(G\left(x_{n}\right)\right)\right)}^{2}.$$

where λ=100 as in the original implementation [39]. Because RegNet is added during training after the generator to account for misaligned targets, the total loss function is defined as

(5) $$L_{Pix2Pix}=L_{Gen}+\beta_{0}L_{Reg}+\beta_{1}L_{Smooth}.$$

where $\beta_{0}=20$ and $\beta_{1}=10$ are the weights for registration loss and smoothing loss, respectively, as suggested by Kong et al. [35].

### 2.5. CycleGAN

CycleGAN [40] has two generators and two discriminators that compete against each other until they converge. Our CycleGAN has a similar architecture of generators and discriminators as Pix2Pix (see Figure 4B). The first generator, $G_{A}$, translates CBCT to sCT, whereas the second generator, $G_{B}$, translates pCT to sCBCT. Similarly, the first discriminator, $D_{A}$, distinguishes whether the sCT is a real or synthetic image, while the second discriminator, $D_{B}$, does the same for sCBCT.

CycleGAN consists of two cycles. In the first cycle, $G_{A}$ translates CBCT to sCT. $G_{B}$ then translates the sCT to cycle CBCT (cCBCT). $D_{A}$ discriminates between cCBCT as a real or synthetic image. In the second cycle, $G_{B}$ then translates CT into sCBCT, and $G_{A}$ then translates sCBCT into cycle CT (cCT). $D_{A}$ discriminates between cCT images as being real or synthetic.

In contrast to the original CycleGAN, which was trained using an unsupervised scheme, we employed supervised learning. As mentioned earlier, our CycleGAN uses similar generator and discriminator losses as Pix2Pix; this is to allow the network to map the image from the source domain to the target domain. In addition, a cycle consistency loss is added to minimize the difference between CT and cCT as well as the difference between CBCT and cCBCT:

(6) $$L_{Cycle}=\frac{1}{N}\sum_{n=1}^{N}|G_{B}\left(G_{A}\left(x_{n}\right)\right)-x_{n}|+\frac{1}{N}\sum_{n=1}^{N}\left|G_{A}\left(G_{B}\left({\tilde{y}}_{n}\right)\right)-{\tilde{y}}_{n}\right|$$

The cycle consistency loss ensures that these mappings are reversed bidirectionally. Similar to Pix2Pix, RegNet is added during training to account for misaligned labels, and the total loss function is then defined as

(7) $$L_{CycleGAN}=L_{Gen_{A}}+L_{Gen_{B}}+\beta_{0}L_{Cycle}+\beta_{1}L_{Reg}+\beta_{2}L_{Smooth}.$$

where $\beta_{0}=10$, $\beta_{1}=20$ and $\beta_{2}=10$ are the weights for the cycle loss, registration loss and smoothing loss, respectively.

### 2.6. UNIT

**Figure 5:**
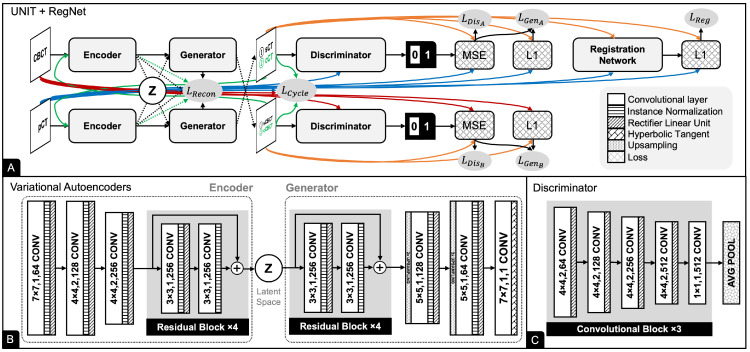
UNIT combined VAEs and GANs for CBCT to CT translation. (**A**) UNIT comprises six subnetworks: two encoders, two generators, and two discriminators. (**B**) The encoder consists of three convolution blocks and four residual blocks to map an image in the source domain into the shared latent code, while the discriminator consists of four residual blocks and three convolution blocks to decode the latent code into an image in the target domain. (**C**) The discriminator consists of a series of convolutions, nonlinear activations, and average pooling operations. Values in the convolutional layer box indicate the size of kernel, the number of strides, and the number of channels, respectively.

UNIT is a framework for image-to-image translation that assumes that a pair of corresponding images in two domains can be mapped to the same latent code in a shared latent space representation z∈Z. UNIT is based on VAE and GAN. It comprises six subnetworks (see Figure 5): two encoders ($E_{A}$ and $E_{B}$), two generators ($G_{A}$ and $G_{B}$), and two discriminators ($D_{A}$ and $D_{B}$). $E_{A}$ first maps CBCT to a code in the latent space $z_{A}\in Z$. $G_{A}$ then decodes the code to reconstruct sCT. Similarly, $E_{B}$ maps CBCT to a code in the latent space $z_{B}\in Z$. $G_{B}$ then decodes the code to reconstruct sCBCT. Similar to Pix2Pix and CycleGAN, the discriminators $D_{A}$ and $D_{B}$ distinguish whether sCT and sCBCT, respectively, are real or synthetic.

Similar to CycleGAN, UNIT also has two GANs. We used a cycle consistency loss:

(8) $$L_{Cycle}=\frac{1}{N}\sum_{n=1}^{N}|G_{A}\left(E_{B}\left(G_{B}\left(E_{A}\left(x_{n}\right)\right)\right)\right)-x_{n}|+\frac{1}{N}\sum_{n=1}^{N}\left|G_{B}\left(E_{A}\left(G_{A}\left(E_{B}\left({\tilde{y}}_{n}\right)\right)\right)\right)-{\tilde{y}}_{n}\right|$$

and a reconstruction loss with L_2_ regularizers:

(9) $$\begin{array}{cc} L_{Recon}= & \lambda \left(\frac{1}{N}\sum_{n=1}^{N}{\left(E_{A}\left(x_{n}\right)\right)}^{2}\right)+\frac{1}{N}\sum_{n=1}^{N}\left|G_{A}\left(E_{A}\left(x_{n}\right)\right)-x_{n}\right|+\\ \; & \lambda \left(\frac{1}{N}\sum_{n=1}^{N}{\left(E_{B}\left({\tilde{y}}_{n}\right)\right)}^{2}\right)+\frac{1}{N}\sum_{n=1}^{N}\left|G_{B}\left(E_{B}\left({\tilde{y}}_{n}\right)\right)-{\tilde{y}}_{n}\right|+\\ \; & \lambda \left(\frac{1}{N}\sum_{n=1}^{N}{\left(E_{B}\left(G_{B}\left(E_{A}\left(x_{n}\right)\right)\right)\right)}^{2}\right)+\lambda \left(\frac{1}{N}\sum_{n=1}^{N}{\left(E_{A}\left(G_{A}\left(E_{B}\left({\tilde{y}}_{n}\right)\right)\right)\right)}^{2}\right). \end{array}$$

where λ=0.001 is a weight for regularization. The reconstruction function encourages a shared latent space to generate corresponding images in the two domains. Coupled with RegNet, the total loss function is defined as

(10) $$L_{UNIT}=L_{Gen_{A}}+L_{Gen_{B}}+\beta_{0}L_{Cycle}+\beta_{1}L_{Recon}+\beta_{2}L_{Reg}+\beta_{3}L_{Smooth}.$$

where $\beta_{0}=10$, $\beta_{1}=10$, $\beta_{2}=20$ and $\beta_{3}=10$ are the weights for cycle loss, reconstruction loss, registration loss and smoothing loss, respectively.

### 2.7. Network Training

This study aimed to address the problem of translating CBCT to CT using deep learning, especially when a model is trained using CBCT and CT acquired at different time points. Thus, it cannot be fully aligned when trained in a supervised manner. RegNet can address this challenge by dynamically correcting the misaligned labels during training. We observed RegNet’s behavior by training Pix2Pix, CycleGAN, and UNIT both with RegNet and without RegNet.

Prior to training, all images have their HU values linearly scaled from [−1024, +3072] to [−1, +1]. We trained each model using the training set and validated it using the validation set. Each model was trained using the Adam optimizer with a learning rate of 0.0001, and a weight decay of 0.0001. Training was performed for 200 epochs, while keeping aside the model with the lowest MAE. We then tested the model with the best performance using the test set. For a fair comparison, we used the same training strategy and hyperparameters for all models. All experiments were run in Pytorch on a workstation with an Intel Core i7-10700 CPU, 64GB RAM, and an NVIDIA Quadro RTX 6000 24GB GPU.

### 2.8. Evaluation Procedures

Our study focused on the image translation task, where the network generates a two-dimensional image. To evaluate the quality of the generated images, we used commonly used metrics, such as mean absolute error (MAE), root mean square error (RMSE), peak signal-to-noise ratio (PSNR), and structural similarity index (SSIM), to compare them with the ground truth images, which are also two-dimensional. We selected these metrics based on a review of similar studies in the literature [27,29,33,35,41], which enables our results to be compared with previous research.

Given that the sCT(x,y) and reference CT(x,y) represent the HU value of location (x,y) of sCT and the corresponding reference CT image, respectively, MAE measures the difference between the two images:

(11) $$MAE=\frac{1}{N_{x}N_{y}}\sum_{x=1}^{N_{x}}\sum_{y=1}^{N_{y}}\left|CT\left(x,y\right)-sCT\left(x,y\right)\right|.$$

RMSE also measures the average errors between the two images, but it gives relatively high weight to large errors:

(12) $$RMSE=\sqrt{\frac{1}{N_{x}N_{y}}\sum_{x=1}^{N_{x}}\sum_{y=1}^{N_{y}}{\left(CT\left(x,y\right)-sCT\left(x,y\right)\right)}^{2}}.$$

SSIM measures the similarity between the two images based on the human visual system:

(13) $$SSIM\left(x,y\right)=\frac{\left(2\mu_{x}\mu_{y}+c_{1}\right)\left(2\sigma_{xy}+c_{2}\right)}{\left(\mu_{x}^{2}+\mu_{y}^{2}+c_{1}\right)\left(\sigma_{x}^{2}+\sigma_{y}^{2}+c_{2}\right)}$$

where $\mu_{x}$, $\mu_{y}$, $\sigma_{x}^{2}$, $\sigma_{y}^{2}$, and $\sigma_{xy}$ are the local means, standard deviations, and cross-covariance for images *x*, *y*. $c_{1}$ and $c_{2}$ are regularization constants.

PSNR represents the ratio between the dynamic range of the image, $MAX_{I}=4096$, and the power of corrupting noise that affects the quality of the image:

(14) $$PSNR=10\log_{10}\left(\frac{{\left(MAX_{I}\right)}^{2}}{\frac{1}{N_{x}N_{y}}\sum_{x=1}^{N_{x}}\sum_{y=1}^{N_{y}}{\left(CT\left(x,y\right)-sCT\left(x,y\right)\right)}^{2}}\right)$$

We observed the performance of each model when both pCT and dCT were used as reference CT. Line profiles of the HU values in both images were plotted to evaluate the images. The resulting sCT was compared with the original CBCT, pCT and dCT using the Hounsfield unit (HU) of regions of interest (ROIs) from different areas. Thirteen 20 cm^2^ square ROIs of were positioned on the sCT and on the corresponding slices and locations of CBCT and pCT.

## 3. Results

All results presented here were obtained from the test set (30 cases). Table 2 shows a comparison of CBCT, pCT, and dCT in terms of different evaluation metrics that could serve as the baselines for our study. dCT is quantitatively more similar to pCT than CBCT. When comparing dCT with CBCT and pCT, MAE decreased from 55.78 to 35.84, RMSE decreased from 148.32 to 118.94, MAE increased from 26.89 to 28.76, and SSIM increased from 0.8168 to 0.8938.

### 3.1. Image Quality Evaluation with Quantitative Metrics

A total of 12 modes were investigated to generate sCT from CBCT. We attempted to explore: (1) the effect of different network architectures, (2) the effect of the registration network, and (3) the effect of using different aligned images (pCT and dCT). Table 3 shows the quantitative evaluation of sCT generated from Pix2Pix, CycleGAN, and UNIT, with and without RegNet, and with pCT and dCT as the target class. The results are expressed in terms of mean and standard deviations, calculated from different slices across different patients. For all modes, the values of MAE and RMSE values calculated from the comparison of sCT with pCT and dCT (see Table 3) were lower than those calculated from the comparison of CBCT with pCT and dCT (see Table 2). The values of PSNR and SSIM calculated from the comparison of sCT with pCT and dCT (see Table 3) were higher than those calculated from the comparison of CBCT with pCT and dCT (see Table 2). We observed that UNIT performed better than CycleGAN and Pix2Pix, that RegNet led to performance improvements in all architectures, and that dCT gave better results than pCT for all modes.

**Figure 6:**
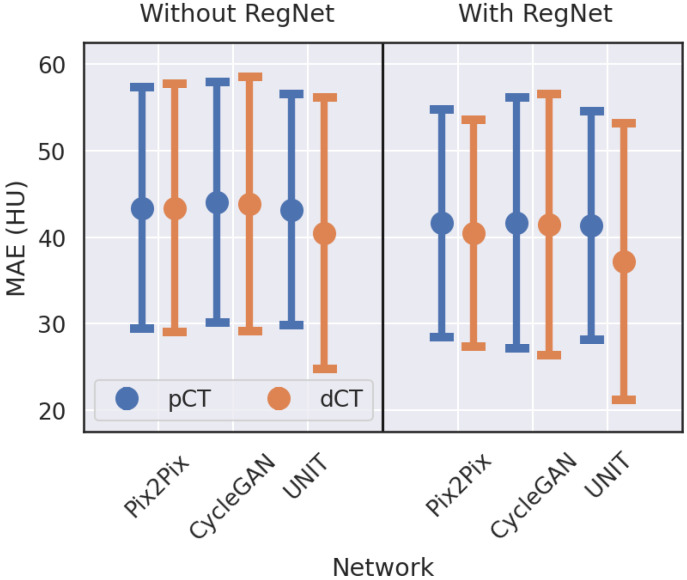
MAEs of sCT generated from different models compared to pCT and dCT. UNIT with RegNet generated sCT with the lowest mean MAE.

Figure 6 illustrates the effects of RegNet on MAEs between sCT generated from different models and from pCT and dCT. For the models without RegNet, UNIT performed slightly better than the other two and dCT resulted in a lower MAE than pCT. For the models with RegNet, the MAEs values were lower for the same configuration and the effects were more pronounced for the models with dCT, especially for UNIT. UNIT with RegNet generated sCT with the lowest mean MAE.

Among the 12 modes, UNIT with RegNet had the highest quantitative performance, with the lowest MAE and RMSE as well as the highest PSNR and SSIM. Thus, this model was adopted in this study.

### 3.2. Image Quality Evaluation in Preserving Anatomy

**Figure 7:**
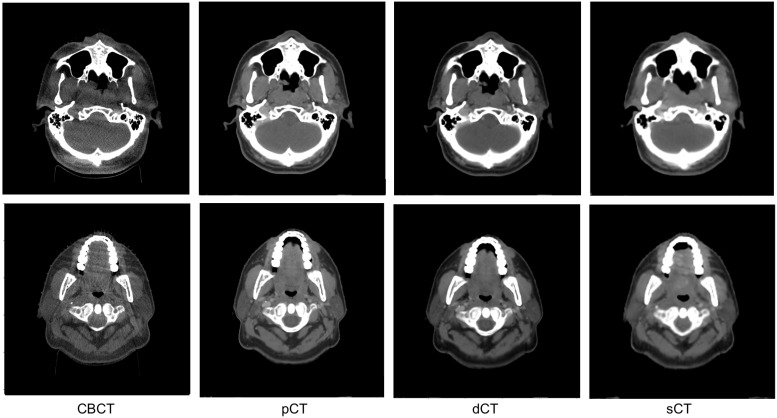
Qualitative comparison of CBCT, dCT, pCT and sCT from two different axial slices in the test set. sCT was generated from the best performing model (UNIT with RegNet).

This study aimed to evaluate not only the accuracy of HU values but also the accuracy of anatomy. Figure 7 shows a comparison of the axial slices of CBCT, pCT, dCT, and the sCT obtained from the proposed model in the head and neck region. CBCT shows artifacts and low contrast resolution, whereas pCT shows clear images with well-detailed image structures. The dCT generated using Velocity^™^ shows organ boundaries as in pCT. The sCT generated from UNIT with RegNet showed reduced scatter artifacts while preserving the anatomy of the original CBCT.

**Figure 8:**
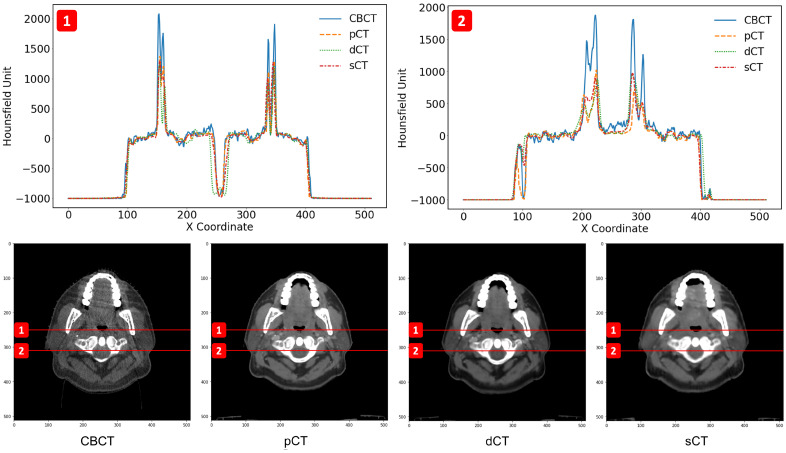
HU line profiles of CBCT, pCT, dCT, and sCT on two different lines passing through the patient’s body in different areas. The top two images show each HU line profile (1 and 2) corresponding to the two red dashed lines (1 and 2) shown in each of the bottom four images of CBCT, pCT, dCT, and sCT.

Figure 8 shows the HU line profiles of the CBCT, pCT, dCT, and sCT images. Two different lines run through the body of the patient in different areas. The top two images show the two HU line profiles (1 and 2) corresponding to the two red dashed lines (1 and 2) seen in the bottom four (CBCT, pCT, dCT, and sCT) images. The top line (1) is plotted at the y-coordinate of 250 and passes through the soft tissue, bone, and air regions. The bottom line (2) is plotted at the y-coordinate of 310 and runs through the soft tissue and bone regions. The line profiles of CBCT illustrate more fluctuations in all areas than in the other images and have a large discrepancy in the bone region compared with the other images. The line profiles of sCT are similar to those of pCT and dCT. However, the dCT deviated slightly in the oropharyngeal regions.

**Figure 9:**
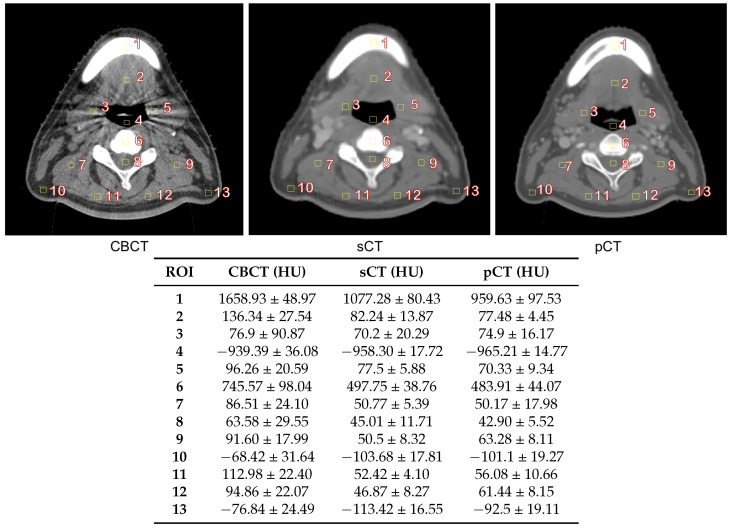
Hounsfield unit (HU) values at different ROIs on CBCT, sCT, and pCT. Values are shown in terms of mean ± standard deviation.

Figure 9 shows the HU values for 13 different regions of interest (ROIs). The ROIs were positioned for bone, air, and soft tissue in different regions on the CBCT, sCT, and pCT images. The HU values of sCT were closer to those of pCT than those of CBCT in all regions.

### 3.3. Patient-Specific Differences

**Figure 10:**
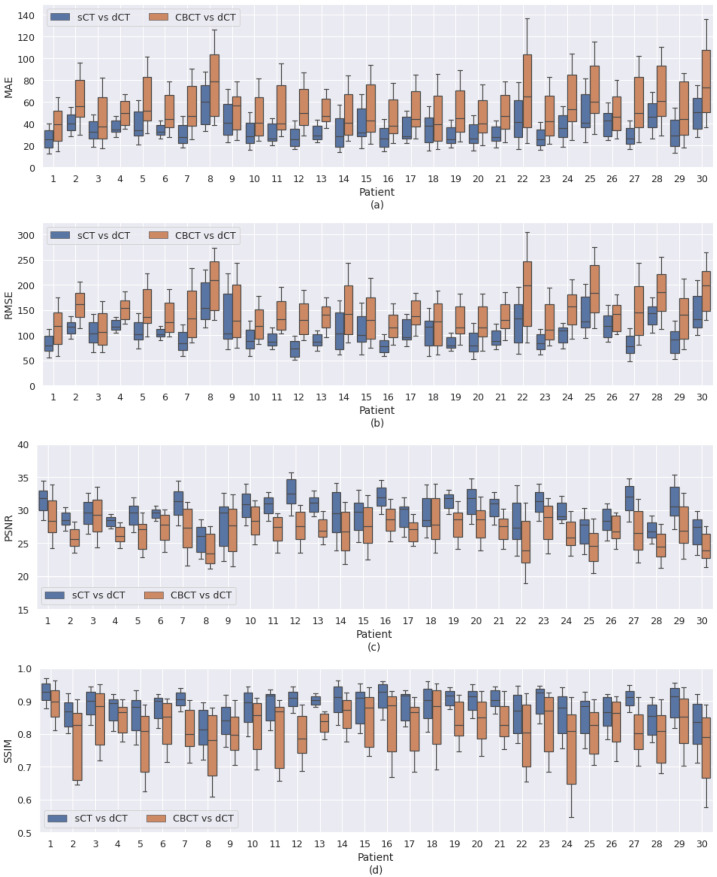
Comparison of sCT with dCT and CBCT with dCT on different similarity metrics, (**a**) MAE, (**b**) RMSE, (**c**) PSNR, and (**d**) SSIM, for each patient in the test set. Values are calculated across all slices from the same patient.

Figure 10 shows a comparison of sCT with dCT and CBCT with dCT in terms of different similarity metrics, such as MAE, RMSE, PSNR, and SSIM, for all patients in the test set. sCT was generated using UNIT with RegNet. The values were calculated across all slices for each patient. This illustrates the variations in the different similarity metrics for each patient. For MAE and RMSE, sCT had lower values than CBCT in all cases. For PSNR and SSIM, sCT had higher values than CBCT in all the cases. The variations in HU values across different patients were not extreme. The results showed that the sCT generated from UNIT with RegNet was improved over the original CBCT.

## 4. Discussion

In this study, we used deep learning to translate CBCT images into CT-like images called sCT. We used several GAN models, namely Pix2Pix, CycleGAN, and UNIT, and coupled them with RegNet during training. RegNet allows supervised learning with slightly noisy labels, which is suitable for our cases because CBCT and CT are usually not acquired simultaneously and there can be a time gap of one week to one month between them. We hypothesized that sCT could be used for accurate dose calculations for adaptive radiotherapy in patients with head and neck cancer.

For our study, we used a dataset of 146 CBCT and CT pairs, with each pair coming from a patient with head and neck cancer. Our dataset contains a wide variety of anatomical structures, which in turn can demonstrate the robustness of GANs to this problem [31,32,33]. In contrast to other studies, we also reported the time gap between the acquisition of pCT and the first fraction of CBCT. We noted that, in our institution, the time gap between CBCT and CT tended to be long, owing to the limited availability of medical resources.

We evaluated the approaches using both pCT (which involved rigid image registration with CBCT) and dCT (which involved deformable image registration with CBCT). All registrations were manually adjusted by clinical experts to best match the CBCT images. We found that the sCT images generated from all GAN models, including Pix2pix, CycleGAN, and UNIT, had better quantitative and qualitative image quality than CBCT in all modes.

The most commonly used architecture for multimodal image translation is CycleGAN for unsupervised learning and Pix2Pix for supervised learning. These architectures are considered state-of-the-art approaches for translating CBCT-to-CT images. However, the quality of the results is highly dependent on the dataset used, and there is no public dataset available. In our experiments using Pix2Pix and CycleGAN, we obtained results that were consistent with those reported in other studies [27,29,33]. Yuan et al. [33] evaluated the image quality of sCT generated from CBCT with U-Net in 55 patients with head and neck cancer and reported the MAE and SSIM values of 49.28 and 0.85, respectively. Maspero et al. [29] trained CycleGAN to generate sCT images using unsupervised training in 15 patients with head and neck cancer. Their results showed that MAE was 51 ± 12. The values observed in the current study differ slightly from those reported by Liang et al. [27], who examined CycleGAN with unsupervised learning and reported lower MAE and RMSE values of 29.85 ± 4.94 and 84.46 ± 12.40, respectively, and higher SSIM and PSNR values of 0.85 ± 0.03 and 30.65 ± 1.36, respectively. These different results may be due to the different image quality of the original CBCT and the reference CT used for comparison.

Our study obtained similar results for the Pix2Pix and CycleGAN models. This could be because both models share common modules, whereas Pix2Pix can be considered a non-cyclic unidirection version of CycleGAN. UNIT, which instead uses a VAE as a generator, outperformed the previous two models. Figure S1 shows the training and validation loss of UNIT. This result agrees with that of Xue et al. [41], who studied patients with nasopharyngeal carcinoma. The authors used CycleGAN, Pix2Pix, and U-Net models to generate sCT from CBCT. They reported that the results using Pix2Pix were comparable to those of CycleGAN, whereas those of U-Net were inferior to those of the other two models. None of the previous studies investigated UNIT with the problem of image translation from CBCT to CT. We observed that UNIT trained with RegNet outperformed all other model variants. We suspect that the shared latent space that reinforces the understanding of the anatomical variations between the two modalities, as used by UNIT, may help to promote the training of paired images of the two modalities.

Our study suggests the use of RegNet in combination with GANs for multi-modal medical image translation. With this approach, RegNet treats imperfectly aligned images as noisy labels, and the training is treated as a supervised learning process with noisy labels. RegNet can effectively compensate for the misaligned noise distribution. The network training aims to find optimal weights for both RegNet and GANs such that it can minimize anatomical changes that occur when source and target images are taken at different time points. This idea is also applicable to other medical images that are taken at different points in time. The benefit of integrating the registration network into the backbone model during training is reflected in Table 3 and Figure 6. RegNet led to performance improvements in all the models, Pix2Pix, CycleGAN, and UNIT. The image quality of CBCT can be significantly improved in the CBCT-to-CT image translation using GANs coupled with RegNet. These findings are consistent with those of other studies [34,35,36,37], which demonstrated that a registration approach can be integrated to the image translation pipeline to improve the performance of the integrated model. The result also supports the use of supervised learning approaches with noisy labels for cases in which perfect alignment of the paired images is not possible. Furthermore, although RegNet is actually a reasonably sized U-Net, it is only coupled with GANs during training, so it does not add to the processing time during inference. In our study, the UNIT with RegNet model demonstrated the best performance, surpassing both Pix2Pix and CycleGAN, which were considered state-of-the-art approaches.

The effect of training the approach with different aligned images (pCT and dCT) is also reflected in Table 3 and Figure 6. The use of pCT, which involves rigid registration with CBCT, may lead to misaligned slices if anatomic structures are changed, especially if the time interval between CT and CBCT is large. On the other hand, the use of dCT, which involves the deformable registration of CT with CBCT, could lead to better structural similarity between the two images. Although neither can represent the real CT, our study suggests that dCT may be more appropriate for the problem because it minimizes the negative effects of anatomical variation. This was supported by the results, which show that sCT generated using dCT gave better results on almost all metrics in our study. While the registration method can effectively minimize unpredictable changes in anatomical tissue position, our results also demonstrate that well-aligned images are generally better suited for training than loosely aligned ones. Similar behavior was reported by Liu et al. [42], who investigated the generation of sCT using CBCT in 30 patients with pancreatic cancer using CycleGANs trained with CBCT against both pCT and dCT. They reported MAE values of 58.45 ± 13.88 and 56.89 ± 13.84 for the model trained with pCT and dCT, respectively. Their results are also consistent with our results.

**Figure 11:**
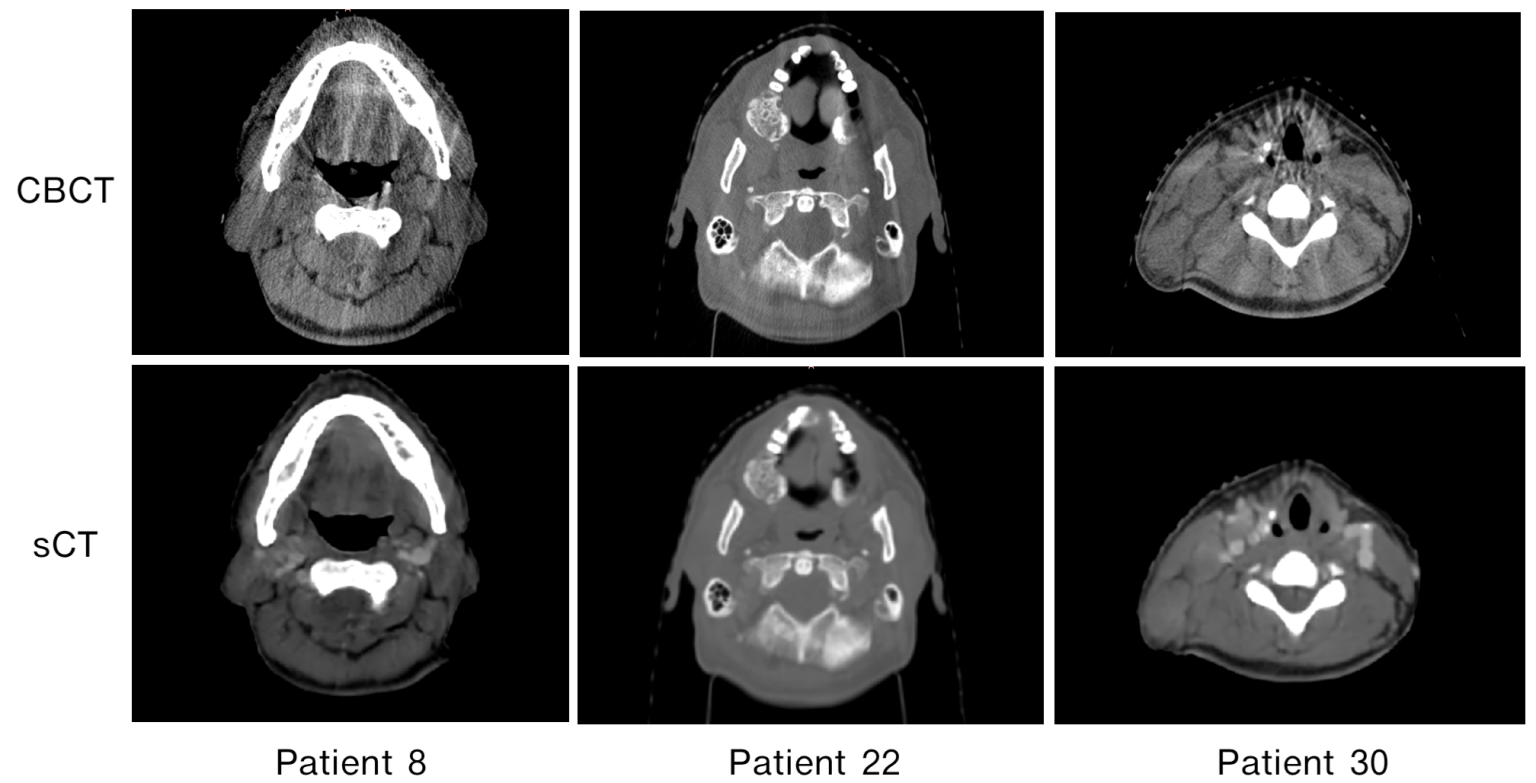
Comparison of CBCT and sCT on axial slices of patients with the three highest average MAEs (Patients 8, 22, and 30).

Figure 7 shows a qualitative comparison of CBCT, dCT, pCT, and sCT obtained using the best-performing model. With sCT, CBCT artifacts in the form of noise and beam hardening (dark streaks) were reduced; however, dental artifacts still appeared. The model did not seems to perform well with large artifacts. In Figure 8, the HU line profiles of the image can be used to compare HU values at each position along the lines and have the potential to evaluate the consistency of the anatomy. The fluctuation of the HU line profiles from CBCT was due to noise, whereas the HU line profiles from dCT were different from those of the other images. It seems possible that these results may be due to the inaccuracy of registration, particularly with the complicated mixing of air and soft tissue [42]. There was an overall improvement of approximately 18 HU between the comparison of dCT and CBCT and the comparison of dCT and sCT. In Figure 9, we can observe that for certain areas, the ROIs of CBCT had higher HU values than those of sCT and pCT. The differences in maximum HU values between CBCT and pCT ranged from bone, soft tissue, fat, and air regions, whereas the differences in HU values of sCT were closer to pCT but differed in certain soft tissue regions. In accordance with our results, a previous study by Kida et al. [43] showed that various artifacts have a stronger effect when spread over the entire CBCT image than in a region near the center of the image. Variations were observed in the similarity metrics of the individual patients in the test set. Figure 10) shows images for patients 8, 22, and 30 as examples. The range of MAE is high and wide in CBCT compared to dCT because of the many artifacts. For sCT, the maximum, minimum, and mean values of MAE reduced from 78.09 to 59.01, 71.91 to 46.70, and 80.06 to 54.85 in patients 8, 22, and 30, respectively. Figure 11 shows the improvement in image quality of sCT compared with the original CBCT.

**Figure 12:**
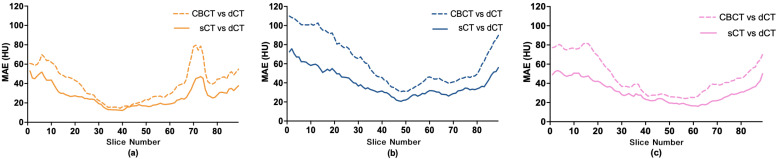
Comparison of sCT with dCT and CBCT with dCT using MAE for each slice on (**a**) Patient 1 (**b**) Patient 5 (**c**) and Patient 10.

The differences in MAE values were observed not only between different patients but also between different slices in the same patient. Figure 12 illustrates the variations in images of three patients from the test set. An interesting result was that the images near the isocenter had a lower MAE than the upper and lower parts of the image volume. These results are probably related to the divergence of the cone beam X-rays, where the peripheral detector receives an X-ray beam with lower energy than the center; therefore, the image quality of CBCT is high near the isocenter.

The deviation between the MAE values of CT with sCT and CBCT is within 50 HU, which is clinically acceptable for a dose deviation of 3% [13]. However, a limitation of this study is the anatomical variation between pCT and the first fraction of CBCT. These anatomical differences may affect network training. In dCT, deformable image registration depends on the image similarity and acquisition quality [44]. Although all registrations were checked and manually adjusted by clinical experts, they still cannot compensate for all alignment discrepancies. Provided that dCT was validated in terms of deformation accuracy, it currently has a higher potential to be used instead of pCT to minimize anatomical variations.

## 5. Conclusions

This study investigated approaches to improve the image quality of CBCT by generating sCT that have the image quality of CT while preserving the patient’s anatomy as in CBCT. We investigated the approaches using paired CBCT-CT imaging data from 146 patients with head and neck cancer. However, these paired data were acquired with different machines and at different time points, with an average interval of two weeks. Therefore, the paired images were not perfectly aligned. We addressed this problem by incorporating RegNet, which can learn the distribution of misaligned noise using GANs during training. RegNet can dynamically estimate the correct labels, enabling supervised learning with noisy labels, whereas GANs learn the bidirectional mapping from CBCT to CT. Using this approach, supervised learning becomes less restrictive and overcomes the limitation that CBCT and CT are not exactly paired. The results presented show that translating CBCT to CT using our approaches can improve the image properties of CBCT to be close to CT. The HU values for sCT are sufficiently accurate to allow for reliable dose calculations, while preserving the anatomy of CBCT with clear structural boundaries. In a future study, we will evaluate the accuracy of sCT dosimetry for clinical use in adaptive radiotherapy.

## Figures and Tables

**Table 1:** Patient characteristics for the training, validation, and test set.

Characteristics	All	Training	Validation	Test
Patients (N)	146	95	21	30
Sex (N)				
− Male	108	68	18	22
− Female	38	27	3	8
Age (Mean ± SD [Range])	54.3 ± 15.2	53.3 ± 15.1	58.4 ± 15.3	54.7 ± 15.4
	[11–89]	[11–87]	[23–85]	[22–89]
Body Mass Index (Mean ± SD [Range])	21.7 ± 4.4	22.2 ± 4.3	20.2 ± 3.9	21.1 ± 4.8
	[12.7–37.9]	[12.7–34.0]	[13.9–30.5]	[15.6–37.9]
Diagnosis site (N)				
− Oral cavity	41	25	8	8
− Nasopharynx	49	41	5	3
− Oropharynx	15	6	3	6
− Hypopharynx	5	2	1	2
− Larynx	10	4	1	5
− Nasal cavity and paranasal sinuses	18	9	3	6
− Salivary gland	3	3	-	-
− Others	5	5	-	-
Pathology results (N)				
− Squamous cell carcinoma	132	81	21	30
− Others	14	14	−	−
TNM classification				
− Primary tumours				
∘ T1	14	11	2	1
∘ T2	28	19	4	5
∘ T3	46	27	7	12
∘ T4	58	38	8	12
− Regional lymph nodes				
∘ N0	30	21	1	8
∘ N1	21	15	3	3
∘ N2	65	38	12	15
∘ N3	30	21	5	4
− Metastasis				
∘ M0	146	95	21	30
∘ M1	−	−	−	−
Treatment				
− Concurrent chemoradiotherapy	108	59	20	29
− Post-operative radiotherapy	26	26	-	-
− Definitive radiotherapy	12	10	1	1
Period between pCT and CBCT in days	16.7 ± 5.4	16.7± 5.4	16.3 ± 5.1	16.8 ± 5.8
(Mean ± SD [Range])	[4–31]	[4–31]	[7–30]	[5–29]

**Table 2:** Comparison of CBCT, pCT, and dCT using different similarity metrics.

Comparison	MAE	RMSE	PSNR	SSIM
**CBCT vs. pCT**	58.16 ± 25.17	160.40 ± 46.08	26.06 ± 2.44	0.8152 ± 0.0859
**CBCT vs. dCT**	55.78 ± 26.15	148.32 ± 51.36	26.89 ± 2.93	0.8168 ± 0.0876
**pCT vs. dCT**	35.84 ± 16.21	118.94 ± 39.30	28.76 ± 2.80	0.8938 ± 0.0483

Values are shown in terms of mean ± standard deviation.

**Table 3:** Quantitative comparison of sCT generated from Pix2Pix, CycleGAN and UNIT, with and without RegNet, and with pCT and dCT as the target class.

Model	Target	RegNet	MAE	RMSE	PSNR	SSIM
**Pix2Pix**	pCT	No	43.39 ± 14.43	134.33 ± 31.40	27.48 ± 1.99	0.8479 ± 0.0542
	pCT	Yes	41.62 ± 13.69	132.00 ± 34.96	27.71 ± 2.32	0.8578 ± 0.0513
	dCT	No	43.34 ± 14.81	133.32 ± 33.68	27.60 ± 2.23	0.8566 ± 0.0515
	dCT	Yes	40.46 ± 13.55	124.07 ± 31.22	28.20 ± 2.12	0.8635 ± 0.0467
**CycleGAN**	pCT	No	44.04±14.44	135.38±32.63	27.43±2.07	0.8480±0.0554
	pCT	Yes	41.67±15.04	131.29±34.07	27.73±2.20	0.8619±0.0549
	dCT	No	43.87 ± 15.23	139.85 ± 35.30	27.18 ± 2.20	0.8527 ± 0.0473
	dCT	Yes	41.44 ± 15.53	124.67 ± 34.43	28.22 ± 2.38	0.8597 ± 0.0591
**UNIT**	pCT	No	43.17±13.82	131.19±31.52	27.70±2.02	0.8517±0.0522
	pCT	Yes	41.34±13.66	128.82±31.78	27.87±2.09	0.8635±0.0518
	dCT	No	40.46 ± 16.21	119.45 ± 37.28	28.67 ± 2.62	0.8630 ± 0.0527
	dCT	Yes	37.21 ± 16.51	108.86 ± 38.13	29.55 ± 2.82	0.8791 ± 0.0547

Values are shown in terms of mean ± standard deviation.

## Data Availability

The data presented in this study are available on request from the corresponding author. The data are not publicly available due to the institutional policy.

## Supplementary Materials

The following supporting information can be downloaded at: https://www.mdpi.com/article/10.3390/cancers15072017/s1, Figure S1: Total loss during training the UNIT model.
