# Supplementary material for: CBCT-to-CT Translation Using Registration-Based Generative Adversarial Networks in Patients with Head and Neck Cancer
Source: Cancers (Basel). 2023 Mar 28;15(7):2017. doi: 10.3390/cancers15072017 (PMC10093508; doi:10.3390/cancers15072017)
Supplement: Supplementary file 1 [file cancers-15-02017-s001.zip › cancers-2263250-supplementary.pdf]

Supplementary File

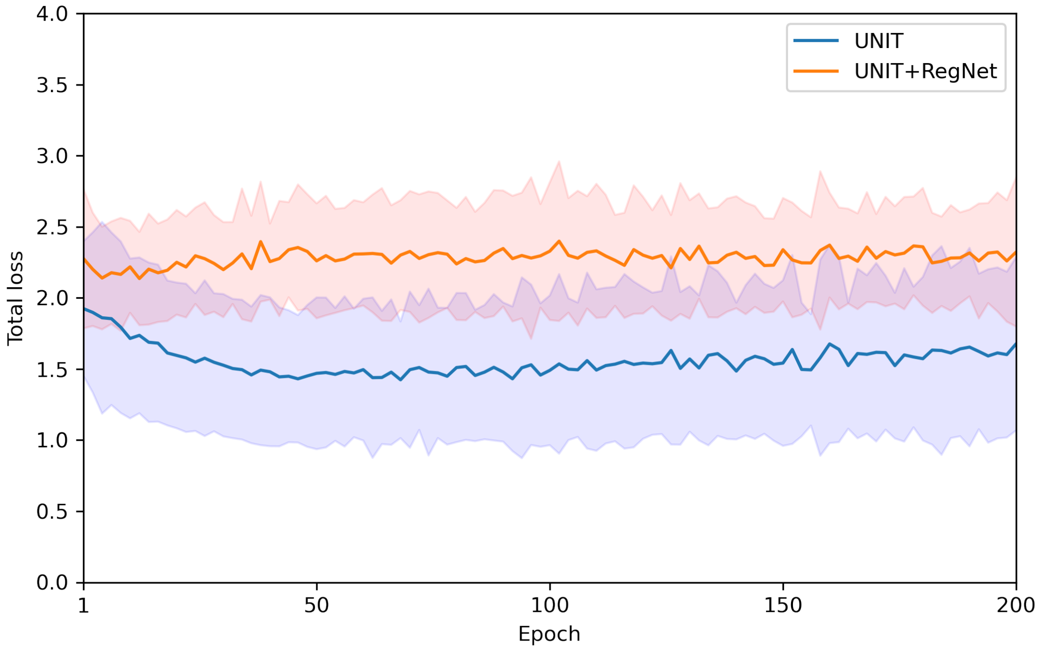

Figure S1: Total loss during training the UNIT model. The loss of the UNIT with RegNet model is higher than that of the UNIT model as its loss function contains the ResNet loss component. We defined the best model as the model from the epoch with the lowest MAE.
